# Left Ventricular Diastolic Dysfunction in a Rat Model of Diabetic Cardiomyopathy using ECG-gated ^18^F-FDG PET

**DOI:** 10.1038/s41598-018-35986-0

**Published:** 2018-12-04

**Authors:** Rudolf A. Werner, Christoph Eissler, Nobuyuki Hayakawa, Paula Arias-Loza, Hiroshi Wakabayashi, Mehrbod S. Javadi, Xinyu Chen, Tetsuya Shinaji, Constantin Lapa, Theo Pelzer, Takahiro Higuchi

**Affiliations:** 1 Department of Nuclear Medicine, University Hospital Wuerzburg, Wuerzburg, Germany; 2 Comprehensive Heart Failure Center, University Hospital Wuerzburg, Wuerzburg, Germany; 3 The Russell H. Morgan Department of Radiology and Radiological Science, Division of Nuclear Medicine and Molecular Imaging, Johns Hopkins University School of Medicine, Baltimore, MD USA; 4 Department of Internal Medicine I, Division of Cardiology, University Hospital Wuerzburg, Wuerzburg, Germany; 5 Department of Biomedical Imaging, National Cardiovascular and Cerebral Research Center, Suita, Japan

## Abstract

In diabetic cardiomyopathy, left ventricular (LV) diastolic dysfunction is one of the earliest signs of cardiac involvement prior to the definitive development of heart failure (HF). We aimed to explore the LV diastolic function using electrocardiography (ECG)-gated ^18^F-fluorodeoxyglucose positron emission tomography (^18^F-FDG PET) imaging beyond the assessment of cardiac glucose utilization in a diabetic rat model. ECG-gated ^18^F-FDG PET imaging was performed in a rat model of type 2 diabetes (ZDF fa/fa) and ZL control rats at age of 13 weeks (n = 6, respectively). Under hyperinsulinemic-euglycemic clamp to enhance cardiac activity, ^18^F-FDG was administered and subsequently, list-mode imaging using a dedicated small animal PET system with ECG signal recording was performed. List-mode data were sorted and reconstructed into tomographic images of 16 frames per cardiac cycle. Left ventricular functional parameters (systolic: LV ejection fraction (EF), heart rate (HR) vs. diastolic: peak filling rate (PFR)) were obtained using an automatic ventricular edge detection software. No significant difference in systolic function could be obtained (ZL controls vs. ZDF rats: LVEF, 62.5 ± 4.2 vs. 59.4 ± 4.5%; HR: 331 ± 35 vs. 309 ± 24 bpm; n.s., respectively). On the contrary, ECG-gated PET imaging showed a mild but significant decrease of PFR in the diabetic rats (ZL controls vs. ZDF rats: 12.1 ± 0.8 vs. 10.2 ± 1 Enddiastolic Volume/sec, P < 0.01). Investigating a diabetic rat model, ECG-gated ^18^F-FDG PET imaging detected LV diastolic dysfunction while systolic function was still preserved. This might open avenues for an early detection of HF onset in high-risk type 2 diabetes before cardiac symptoms become apparent.

## Introduction

As one of the most devastating diseases, diabetes mellitus (DM) type 2 is also called “The Epidemic of the Century”^1^: Mainly due to an increased awareness of physicians and better diagnostic tests, the rates of diabetes-associated complications have declined. However, epidemiologists forecast that its prevalence will rise up to 33% in the year 2050^2^ and therefore, an extensive disease burden can be anticipated in both the United States and Europe^2,3^. The Framingham Study has intensively reported on an increased risk of heart failure (HF) development prior to clinical manifestation of DM^4^ and consequently, novel non-invasive diagnostic strategies to reveal an early onset of HF in diabetic cardiomyopathy (CM) are intensively sought.

Diastolic HF is defined as an increased filling pressure that is needed to achieve ventricle filling to a normal end-diastolic volume, while left ventricular (LV) systolic function is not hampered^5^. As a clinical impetus, diastolic dysfunction has attracted interest, mainly due to its presence in asymptomatic DM patients and as an early sign of a chronic heart disease, such as diabetic CM^6,7^. The latter one is defined as structural and functional heart abnormality in DM patients that is not directly attributable to another underlying pathological cause (e.g. coronary artery disease or arterial hypertension)^8^. Of note, impaired diastolic function is a common phenomenon in asymptomatic patients suffering from diabetes and therefore, it should be assessed systematically among those high-risk patients^9^.

In recent years, a large variety of animal models have been investigated to study diabetic CM and its underlying pathology, in particular by using echocardiography or magnetic resonance imaging (MRI) for the evaluation of both LV systolic and diastolic function^10^. Apart from that, electrocardiography (ECG)-gated ^18^F-fluorodeoxyglucose positron emission tomography (^18^F-FDG PET) has been established for the assessment of glucose utilization of the heart, as well as LV volumes and LV ejection fraction (LVEF) in clinical PET studies^11–13^. However, its potential for the assessment of ventricular performance in a dedicated diabetic CM small animal model has not been investigated yet.

Therefore, we aimed to elucidate the capability of ECG-gated ^18^F-FDG PET in the detection of LV diastolic dysfunction in a rat-model of type 2 diabetes.

## Material and Methods

### Animal Model

Animal protocols were approved by the local Animal Care and Use Committee (Regierung von Unterfranken, Germany) and conducted according to the Guide for the Care and Use of Laboratory Animals^14^. Zucker lean (ZL) controls rats and Zucker diabetic fatty (ZDF *fa/fa*) rats (Charles River, Wilmington, n = 6, respectively) were investigated at an age of 13 weeks (ZL controls vs. ZDF rats, body weight, 290 ± 15 g vs. 382 ± 55 g, p < 0.01).

### Small-animal PET and Imaging Protocol

Fasting was performed >10 h (ZL controls vs. ZDF rats, fasting glucose levels prior to the scan, 113.7 ± 16.2 vs. 239 ± 85.2 mg/l, P < 0.01). A high resolution dedicated small animal PET system (Inveon micro PET, Siemens Medical Solutions Inc., Erlangen, Germay) was used for data acquisition. Its specifications have been described previously^15^. ^18^F-FDG was synthesized in-house according to the manufacturer’s instructions. All animals were maintained under anesthesia throughout the imaging procedure. To enhance cardiac activity, approximately 37 MBq ^18^F-FDG were administered via the tail vein under hyperinsulemic-euglycemic clamp^16^. A 35-min list-mode PET acquisition with ECG recording was started shortly before tracer injection. The data were sorted into 3-dimensional sonograms of 16 frames per cardiac cycle, which were then rebinned with a Fourier algorithm to reconstruct dynamic images using a 2-dimensional ordered-subset expectation maximization method. All reconstructed images were corrected for ^18^F decay, random coincidences, and dead time. A 13 min transmission scan was also conducted prior to the emission scan for attenuation correction^17^.

### PET Data Analysis

**Figure 1:**
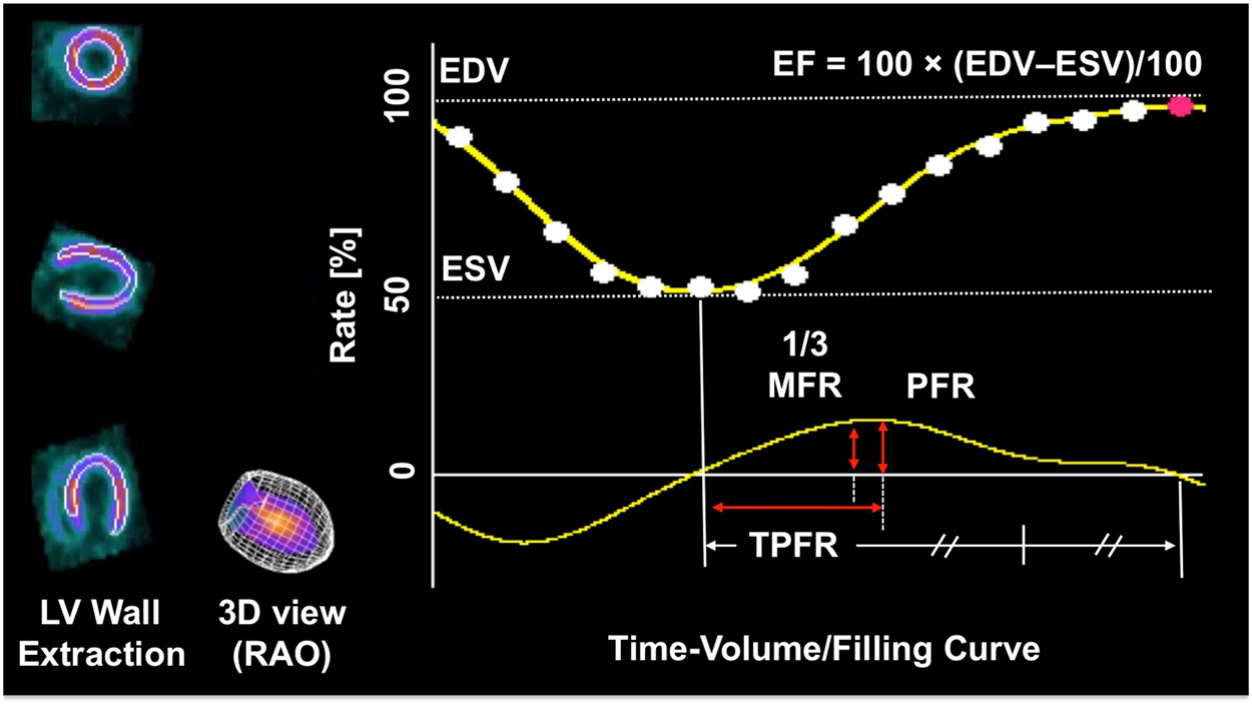
Time volume/filling curve. Including systolic (Ejection fraction, EF) and diastolic parameters (One-third mean filling rate (1/3MFR), Peak Filling Rate (PFR), Time to PFR (TPFR)). EDV = End-diastolic Volume. ESV = Endsystolic Volume. RAO = right anterior oblique view.

For LV functional and volume analysis, image data from 15 to 35 min after tracer injection were employed. Evaluation of LV function and volume was performed using a dedicated automatic ventricular edge detection software (Heart Function View, Nihon Medi-Physics Co. Ltd., Tokyo, Japan), which had been adapted to the size of a rat heart (5 fold magnification of the voxel size of the reconstructed images). The following parameters were assessed using a programme feature for 16-gated myocardial perfusion single photon emission computed tomography: As volume parameters, end-diastolic and end-systolic volumes (EDV and ESV, microliter). As a systolic parameter, ejection fraction (EF, defined as the % of the stroke volume compared to the EDV). As diastolic parameters, the peak filling rate (PFR, defined as the maximum dV/dt value divided by EDV, per second), the one-third mean filling rate (1/3MFR, defined as the average of dV/dt values in the first third of the filling time, per second) and the time to PFR (TPFR, defined as the time from end-systole to PFR, per millisecond) were investigated (Fig. 1)^18,19^. Heart Rate (HR) was also compared.

### Statistical Analysis

All results are displayed as mean ± standard deviation. The two-tailed paired Student’s t-test was used to compare differences between two dependent groups, and the two-tailed independent Student’s t-test for differences between independent groups. The use of the latter test has been recommended, in particular for smaller sample sizes^20^. A P-value of less than 0.05 was assumed to be statistically significant. Statistical analysis was done with StatMate III (ATMS Co., Ltd).

## Results

### LV Functional and Volume Assessment

**Figure 2:**
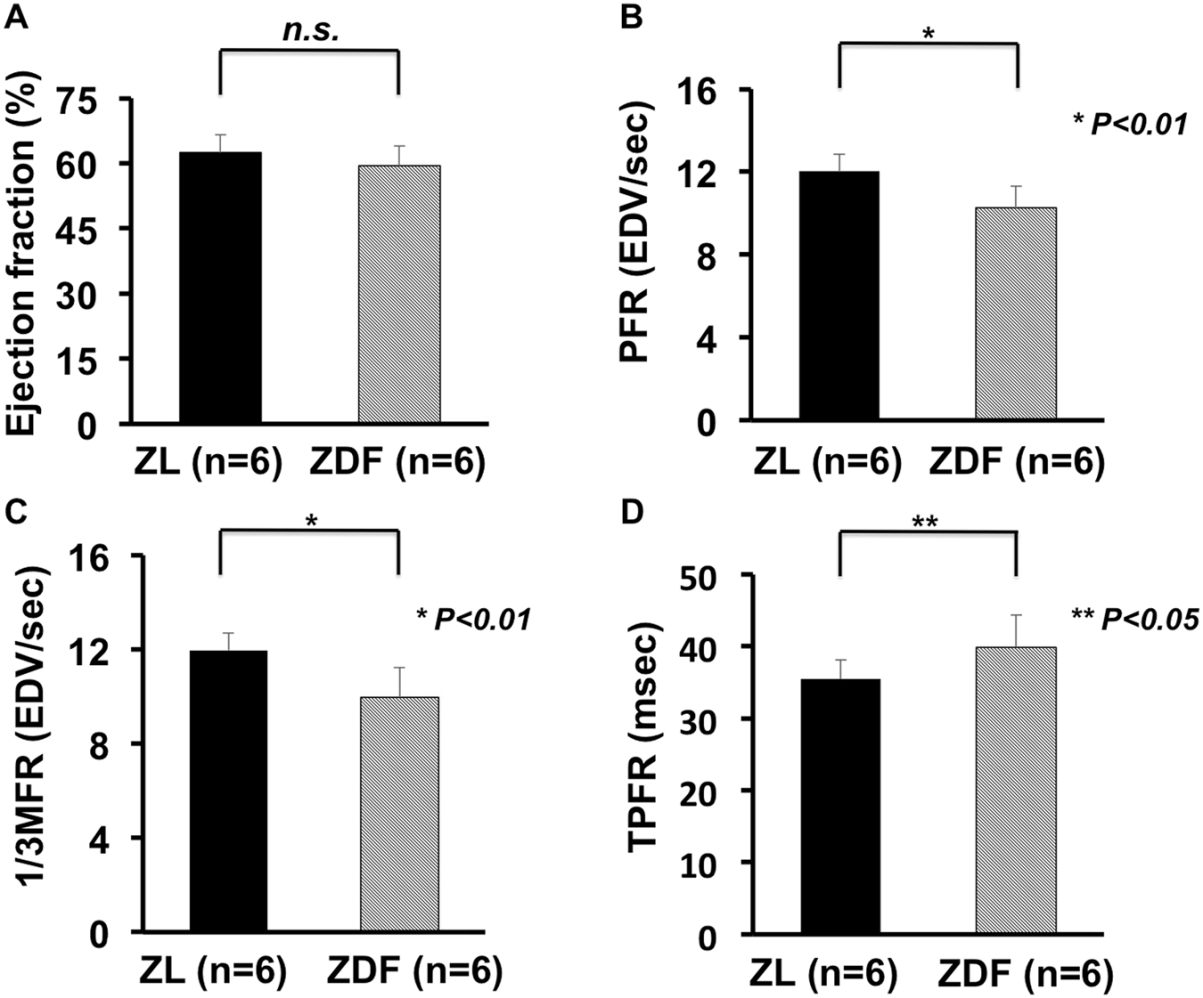
Left ventricular (LV) functional assessment using ECG-gated 18F-FDG PET. (**A**) Left ventricular Ejection fraction (EF) demonstrated no significant difference between the ZDF rat model and ZL controls. On the contrary, diastolic parameters revealed significant differences between both groups. (**B**) Peak Filling Rate (PFR). (**C**) One-third mean filling rate (1/3MFR) and (**D**) Time to PFR (TPFR)). Hence, diastolic dysfunction with preserved systolic function could be proven in ZDF rats. (Zucker lean (ZL) controls rats and Zucker diabetic fatty (ZDF) rats: n = 6, respectively).

ECG-gated PET in the ZDF rat model indicated a reduced diastolic function, while the systolic function was still preserved: No significant difference in the systolic assessment could be obtained (ZL controls vs. ZDF rats: LVEF, 62.5 ± 4.2 vs. 59.4 ± 4.5%, n.s., Fig. 2A). On the contrary, PFR assessed in diabetic rats revealed a mild but significant decrease (ZL controls vs. ZDF rats: PFR, 12.1 ± 0.8 vs. 10.2 ± 1 EDV/sec, P < 0.01, Fig. 2B). Moreover, the 1/3MFR and TPFR also both differed significantly (ZL controls vs. ZDF rats: 1/3MFR, 12.0 ± 0.7 vs. 9.9 ± 1.2 EDV/sec, P < 0.01; and TPFR, 35.4 ± 2.7 vs. 40.0 ± 4.2 msec, P < 0.05; Fig. 2C,D, respectively). HR, EDV and ESV also did not differ significantly (ZL controls vs. ZDF rats: HR, 331 ± 35 vs. 309 ± 24 bpm; EDV, 410.8 ± 60.3 vs. 478.8 ± 77.9 µL; ESV, 155.1 ± 31.4 vs. 194.1 ± 36.6 μL, n.s., respectively).

## Discussion

Investigating a dedicated diabetic CM rat model with micro PET, diastolic function was significantly altered, while systolic performance was still preserved. Hence, in a tailored treatment approach, multiparametric ^18^F-FDG PET assessment might pave the way to detect an early onset of cardiac involvement in DM patients: Consequently, even in the absence of clinically apparent signs of HF, promotion of lifestyle changes for the prevention of diabetes-associated cardiac diseases could be intensified^21^ or treatment at an earlier time point could be initiated. Thus, multiparametric ^18^F-FDG PET might have the potential to advance personalized treatment in high-risk DM patients.

To assess the clinical condition of diastolic HF, left-sided heart catherization may give ultimate evidence, but its invasive nature may limit its widespread adoption^22^. Thus, several non-invasive imaging modalities have been advocated to reliabely investigate the phenomenon of diastolic dysfunction. However, all of those imaging modalities may have some drawbacks: For instance, MRI has a rather limited resolution, in particular when compared to transthoracic echocardiography (TTE). In addition, cardiac MRI cannot be routinely performed in patients with limiting preconditions, such as subjects with implantable devices or rhythm disorders^23^. As a fast, accurate and easily available method, TTE is extensively performed to assess this phenomenon among high-risk individuals, but it may be limited by its acoustic window^22,23^. In addition, paroxysmal or permanent atrial fibrillation may also limit its interpretation^24^. Thus, novel non-invasive imaging modalities may overcome those hurdles and ^18^F-FDG PET may serve as an attractive alternative, e.g. by investigating subclinical diastolic dysfunction with preserved EF in patients with implantable devices.

Previous studies have reported on the use of imaging tests in dedicated diabetes small-animal models: Assessing cardiac function via echocardiography, *Marsh et al*. demonstrated that Zucker diabetic fatty obese rats suffered from impaired LV relaxation (along with increased arterial stiffness)^25^. In a previous investigation also relying on echocardiographic images, a dilated ventricle with reduced subnormal contraction was proven in a head-to-head comparison between ZDF *fa/fa* rats and ZL controls^26^. In addition, ^18^F-FDG PET studies for the assessment of LV function have also been performed previously in healthy animals: Investigating MRI as a reference standard in healthy mice, an LV function obtained by ^18^F-FDG PET demonstrated an excellent correlation compared to MRI^27^. Similar results could be obtained for healthy rats: in a comparison of ^18^F-FDG PET with a clinical MRI system, LVEF values were almost identical to the herein obtained LVEF values of ZL control rats^28^. In an elegant approach, *Todica et al*. recently reported on the use of the novel blood-pool tracer ^68^Ga-albumin in healthy Sprague-Dawley rats: notably, compared to the gold standard MRI, an even better correlation between LVEF values could be obtained for ^68^Ga-albumin than for ^18^F-FDG^29^. Moreover, *Aikawa* and coworkers recently investigated ^11^C-hydroxyephedrine (^11^C-HED) in HF patients with preserved ejection fraction: altered myocardial sympathetic innervation was associated with the presence of advanced diastolic dysfunction^30^. Hence, other promising cardiac PET imaging agents could potentially be applied in the herein described diabetes rat model and might pave the way for further insights into diabetic CM prior to the onset of cardiac involvement in the long run.

Apart from that, the age of the investigated ZDF rats is of utmost importance: analogous to our study, *Welch et al*. studied ZDF rats at week 12, which is considered to be consistent with the metabolic alterations occurring at an early stage of type 2 DM in human subjects^31,32^. To further strengthen our preliminary findings, we also investigated ^18^F-FDG under hyperinsulinemic-euglycemic clamp conditions: This strategy is known to provide an excellent image quality and is superior to other conventional techniques to enhance cardiac activity, such as glucose load and/or insulin bolus prior to image acquisition^33–35^.

This study has several limitations: First, it is discussed controversially which animal model may fulfil all disease conditions present in a human setting and patients suffering from DM. Thus, other animal models could also be investigated, e.g. *db/db* mice and *ob/ob* mice^36^. Nonetheless, given a potential partial volume effect on PET, the larger size of a rat heart may be more suitable in the present study design^37^. In addition, such examinations in ZDF/ZL rats could also be conducted in recently introduced imaging modalities, such as (PET/)MRI or with the more commonly used TTE^38^. In particular, the latter imaging technique could also serve as a gold standard, in a manner similar to left-sided heart catherization^22,23^.

## Conclusions

In a diabetic CM rat model under insulin clamp, ECG-gated ^18^F-FDG PET demonstrated distinguished characteristics to assess impaired diastolic function, while systolic function was not significantly altered. Hence, reflecting a typical pathological condition which occurs early in diabetic CM prior to symptomatic manifestation of cardiac involvement, this novel PET-based strategy might set the scene for identifying high-risk patients before cardiac symptoms become clinically apparent. In light of the results of the present study, future efforts may focus on investigating this phenomenon in a clinical setting, e.g. by performing PET studies in DM patients at an early stage of disease. This could be done not only with the”working horse” in PET imaging, namely ^18^F-FDG, but also with other, recently emerging cardiac radiotracers, such as the blood-pool tracer ^68^Ga-albumin, or with imaging probes evaluating cardiac innervation (^11^C-HED or ^18^F-LMI1195)^29,30,39,40^. Such an approach of a global assessment of different cardiac conditions may pave the way to obtain further insights in the underlying pathology of diastolic dysfunction in DM patients.

## Data Availability

The datasets generated during and/or analysed during the current study are available from the corresponding author on reasonable request.
